# Matrix Metalloproteinase-1 and Matrix Metalloproteinase-9 in the Aqueous Humor of Diabetic Macular Edema Patients

**DOI:** 10.1371/journal.pone.0159720

**Published:** 2016-07-28

**Authors:** Jin-woo Kwon, Jin A. Choi, Donghyun Jee

**Affiliations:** Department of Ophthalmology and Visual Science, St. Vincent’s Hospital, College of Medicine, Catholic University of Korea, Suwon, Korea; University of Patras, GREECE

## Abstract

**Purpose:**

To assess the concentrations of matrix metalloproteinase (MMP)-1 and MMP-9 in the aqueous humor of diabetic macular edema (DME) patients.

**Method:**

The concentrations of MMP-1 and MMP-9 in the aqueous humors of 15 cataract patients and 25 DME patients were compared. DME patients were analyzed according to the diabetic retinopathy (DR) stage, diabetes mellitus (DM) duration, pan-retinal photocoagulation (PRP) treatment, recurrence within 3 months, HbA1C (glycated hemoglobin) level, and axial length.

**Results:**

The concentrations of MMP-1 and MMP-9 of the DME groups were higher than those of the control group (p = 0.005 and p = 0.002, respectively). There was a significant difference in MMP-1 concentration between the mild non-proliferative diabetic retinopathy (NPDR) group and the proliferative diabetic retinopathy (PDR) group (p = 0.012). MMP-1 concentrations were elevated in PRP-treated patients (p = 0.005). There was a significant difference in MMP-9 concentrations between the mild NPDR group and the PDR group (p < 0.001), and between the moderate and severe NPDR group and the PDR group (p < 0.001). The MMP-9 concentrations in PRP treated patients, DM patients with diabetes ≥ 10 years and recurrent DME within 3months were elevated (p = 0.023, p = 0.011, and p = 0.027, respectively). In correlation analyses, the MMP-1 level showed a significant correlation with age (r = -0.48, p = 0.01,), and the MMP-9 level showed significant correlations with axial length (r = -0.59, p < 0.01) and DM duration (r = 049, p = 0.01).

**Conclusions:**

Concentrations of MMP-1 and MMP-9 were higher in the DME groups than in the control group. MMP-9 concentrations also differed depending on DR staging, DM duration, PRP treatment, and degree of axial myopia. MMP-9 may be more important than MMP-1 in the induction of DM complications in eyes.

## Introduction

Diabetic retinopathy (DR) is one of the most important causes of visual impairment in many developed countries, despite advances in laser and surgical treatments[1–3]. Based on studies of vascular endothelial growth factor (VEGF) and anti-VEGF antibody treatments for DR, several types of anti-VEGF antibodies have been used and found effective for this disorder[4, 5]. However, recent studies have reported that levels of not only VEGF but also transforming growth factor, epidermal growth factor, human growth factor, interleukins, intercellular adhesion molecule-1, interferon gamma–induced protein, monocyte chemoattractant protein, matrix metalloproteinases (MMPs), plasminogen activator inhibitor-1, placenta growth factor, and tissue growth factor-beta are elevated in the vitreous and anterior chambers[6–8]. Their roles in DR may be associated with alterations in the blood-retinal barrier (BRB), although their exact mechanisms of action remain unclear[7, 8].

The MMPs comprise a group of zinc- and calcium-dependent endopeptidases that are involved in physiological and pathological processes associated with extracellular matrix (ECM) remodeling. MMPs are involved in the degradation and re-building of ECM proteins such as collagen, elastin, gelatin, and casein[9]. At least 25 MMPs have been identified and separated by function into collagenases (MMP-1, MMP-8, and MMP-13), gelatinases (MMP-2 and MMP-9), stromelysins (MMP-3, MMP-10, and MMP-11), matrilysins (MMP-7 and MMP-26), membranous MMPs (MMP-14, MMP-15, MMP-16, MMP-17, MMP-24, and MMP-25), and others[10]. Previous studies have reported that levels of MMPs may be associated with macro- and microvascular complications in DM [11–17].

The associations between retinal vascular complications, MMPs, and DM remain under investigation. Recently, some studies of PDR patients reported increased levels of certain MMPs in the vitreous[18–21]. Such up-regulation of MMPs may involve PDR angiogenesis and progression via degradation of the basement membrane and the ECM of the retina, featuring release of VEGF from ECM-associated reservoirs[22, 23]. However, the roles played by specific MMPs in DR progression remain to be investigated. In previous studies, MMP-1 and MMP-9 were the factors considered most likely to be associated with development of DR complications [8, 20, 24, 25]. Although MMP-1 is well-known as a collagenase and MMP-9 is well-known as a gelatinase, the roles of these proteinases in DR and in alterations of the BRB remain unclear [16, 26, 27].

As reported in most previous studies, vitreous samples yield optimal information on the status of the retina, but obtaining such samples is unacceptably invasive and data quality may be compromised if vitreous samples are mixed with blood (from a hemorrhage). After anti-VEGF agents have been extensively applied, the requirement for vitrectomy decreases. In addition, some studies evaluating MMPs in aqueous humor have shown that the data obtained are valuable[8, 25].

We hypothesize that MMP-1 and MMP-9 have major roles in the initiation of inner BRB damage and in the induction of diabetic macular edema (DME). This study therefore evaluated the concentrations of MMP-1 and MMP-9 in the aqueous humor of DME patients.

## Methods

This study compared the concentrations of MMP-1 and MMP-9 in the aqueous humors of cataract patients and DME patients with type II diabetes mellitus (DM), and followed the tenets of the Declaration of Helsinki. The protocol was approved by the Institutional Review/Ethics Board of the Catholic University, Korea. All participants gave written informed consent for the use of their clinical records in this study.

### Study subjects

Eyes with DR with a macula thickness > 300 μm due to DME were included in the study. Exclusion criteria included eyes with glaucomatous disc changes, retinal degeneration, and macular edema due to other causes. Eyes with concurrent diseases such as age-related macular degeneration, epiretinal membrane, and retinal vascular occlusion, as well as eyes with histories of severe ocular trauma, uveitis, or prior intraocular surgery that could influence the enzyme levels of the aqueous humor were also excluded.

All patients underwent a full ophthalmological examination that included measurement of visual acuity, refraction, and axial length (if checkable), intraocular pressure (IOP) using Goldmann applanation tonometry, a dilated fundus examination, and fluorescein angiography to classify the eyes according to the Early Treatment of Diabetic Retinopathy Study as mild non-proliferative diabetic retinopathy (NPDR), moderate and severe NPDR, or proliferative diabetic retinopathy (PDR). The ophthalmological examination also included assessment of macular thickness using optical coherence tomography (OCT) (Cirrus High Definition OCT; Carl Zeiss Meditec, Dublin, CA, USA), and axial length using the IOL Master (Carl Zeiss Meditec).

### Assay of MMP-1 and MMP-9

The concentrations of MMP-1 and MMP-9 in the aqueous humor in the anterior chamber were determined using anterior paracentesis during cataract surgery. The volume of the collected aqueous humor was at least 50 μL. Human MMP antibody- immobilized beads and 25 μL of 1:20 diluted samples were used. We incubated 2 hours at room temperature (20–25°C) after adding mixed beads, and 1 hour room temperature after adding detection antibodies, and 30 minutes at room temperature after adding Streptavidin-Phycoerythrin. Samples were read using Luminex x-MAP^®^ suspension array technology (Austin, TX, USA). We employed a multiplexed microsphere suspension immunoassay that detects and quantitates spectrally unique microspheres attached to specific antibodies. This enables analysis of samples from a large number of subjects in a single reaction [28, 29]. The detection limit for analysis was 0.36 pg/mL, with a dynamic range of up to 10,000 pg/mL for MMP-1, and 8.71 pg/mL with a dynamic range of up to 10,000 pg/mL for MMP-9. All values under the lower limit of detection were considered zero values[8, 30, 31].

### Statistical treatment of data

Statistical analyses were performed using SPSS for Windows software (ver. 20.0; SPSS, Chicago, IL, USA) and R (ver. 3.2.3) [2015-12-10, Platform: x86_64-redhat-linux-gnu, R Core Team (2015). R: A language and environment for statistical computing. R Foundation for Statistical Computing, Vienna, Austria.URL https://www.R-project.org/.]

The DME and control groups were compared using the nonparametric Mann–Whitney U test and the chi-square test. We conducted DME subgroup analyses of MMP-1 and MMP-9 concentrations by reference to pan-retinal photocoagulation (PRP) status, diabetes duration (< 10years versus ≥ 10years), and recurrence within 3 months, using a nonparametric Mann–Whitney U test. Subgroups created by DR staging were classified as having mild, moderate and severe NPDR, or PDR, and were compared using the Kruskal-Wallis test. Tukey’s method was used to compensate for multiple statistical analyses and comparisons as post hoc analyses. Spearman’s correlation test was used in subgroup analyses to evaluate the correlations between age, duration of DM, the level of HbA1C (glycated hemoglobin), and axial length, and the concentrations of MMP-1 and MMP-9. All data in the text are presented as averages ± standard deviations.

## Results

We enrolled 25 DME patients as a study group and 15 cataract patients as a control group. Refraction and axial length were checked in 17 and 23 patients of the DME group, and 14 and 15 patients of the control group, respectively. The average age of the cataract patients was 63 ± 15 years and that of the DME patients was 65 ± 13 years (p = 0.804). There were 12 males and 13 females in the study group and 9 males and 6 females in the control group. There were no significant differences in spherical equivalent, IOP, or axial length between the study and control groups (Table 1).

**Table 1:** Demographics and baseline clinical characteristics of the study participants.

	Control group (n = 15)	DME group (n = 25)	p value
Age (years)	63 ± 15	65 ± 13	0.804
Sex (male: female)	9:6	12:13	0.342
IOP (mmHg)	12± 4	13 ± 3	0.192
Spherical equivalent (diopters)	0± 2 (n = 14)	0 ± 2 (n = 17)	0.710
Axial length (mm)	24± 1 (n = 15)	23 ± 1 (n = 23)	0.068

IOP, intraocular pressure; DME, diabetic macular edema.

### Comparison of DME and control group

**Fig 1:**
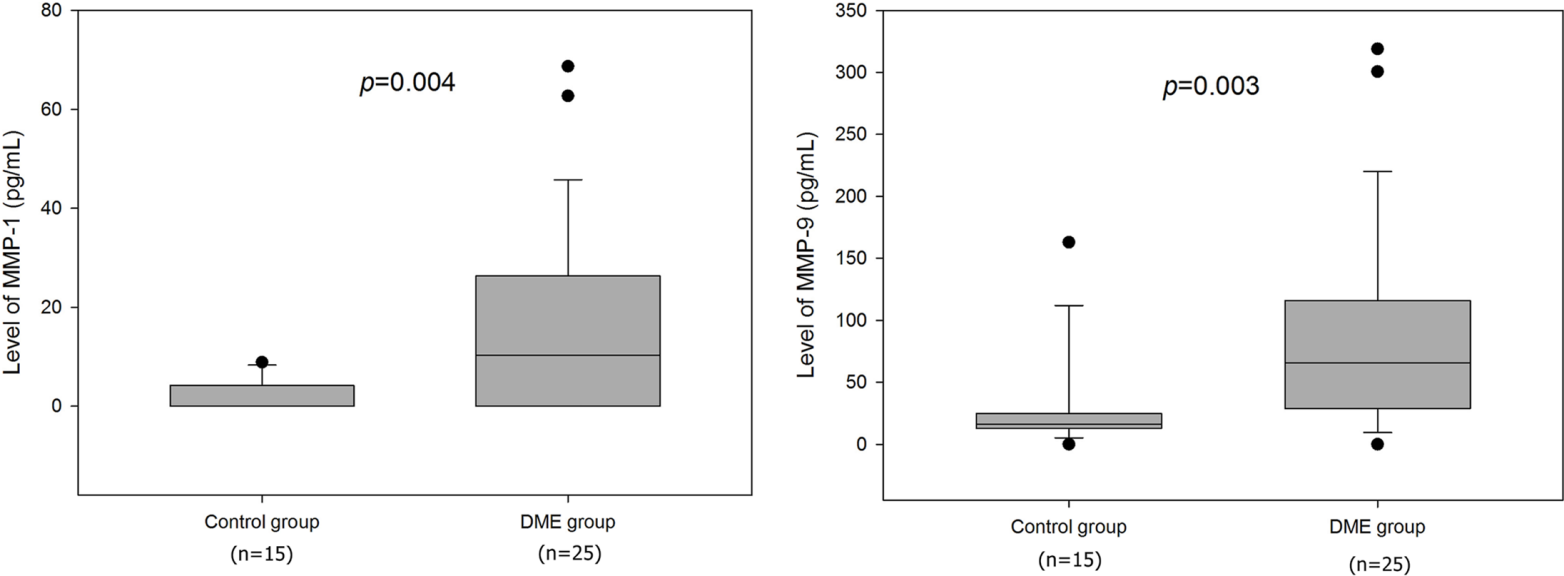
The box and whisker plots for levels of metalloproteinases (MMPs). The bottom and top of the box are the first and third quartiles with a line to show the median value. The whiskers show the 10^th^ and 90^th^ percentiles. Circles represent outliers. The levels of MMP-1 and MMP-9 are increased in diabetic macular edema (DME) group with significant differences.

We found a significant difference between the average MMP-1 concentrations in the study group (16 ± 19 pg/mL) and those in the control group (2 ± 3 pg/mL, p = 0.005). The average concentration of MMP-9 also differed significantly between the study group (84 ± 80 pg/mL) and the control group (31± 41 pg/mL, p = 0.002). The p-values in the box plots where median values were compared are slightly different from p-values in the text where mean values were compared. However, statistical significances were not different (Fig 1).

### DME subgroup analysis by the stage of DR

The concentrations of MMP-1 and MMP-9 by the stage of DR also differed significantly among groups (p = 0.03, p<0.001, respectively). Post hoc analysis showed a significant difference in MMP-1 concentrations between the mild NPDR group (n = 8, 5 ± 6 pg/mL) and the PDR group (n = 9, 29 ± 24 pg/mL, p = 0.012). We found no significant differences between the mild NPDR group and the moderate or severe NPDR group (n = 9, 11 ± 13 pg/mL) (p = 0.351), or between the moderate or severe NPDR group and the PDR group (p = 0.077).

**Fig 2:**
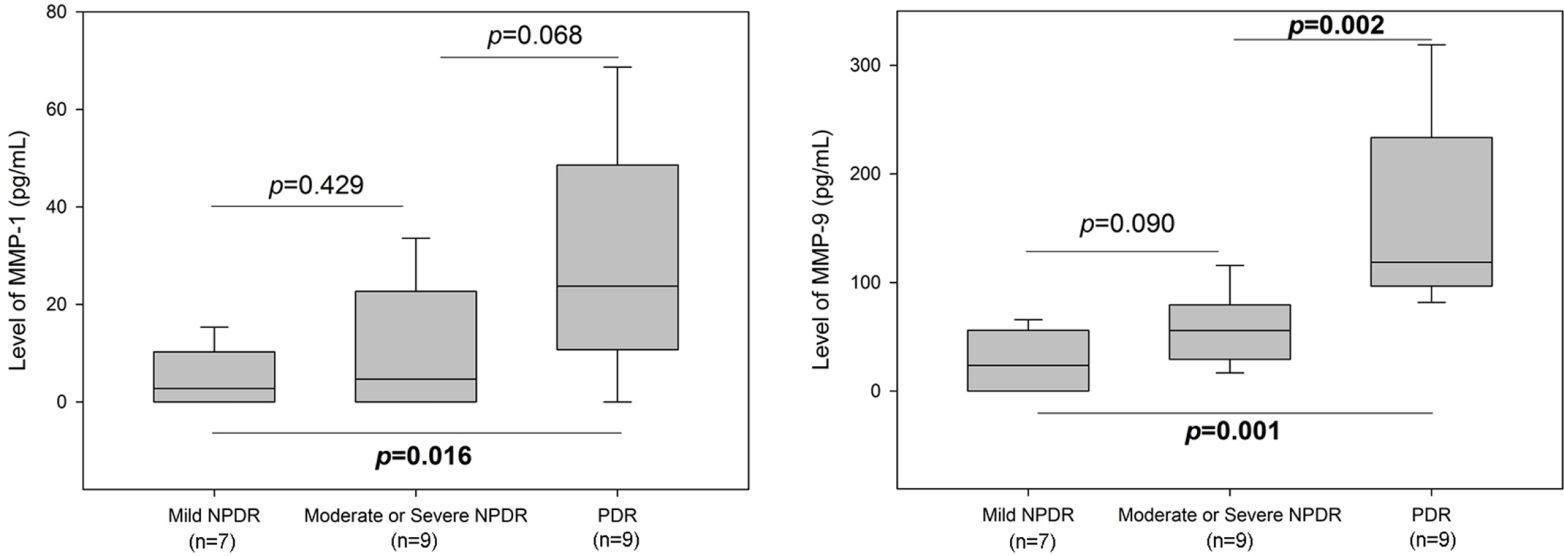
The concentrations of metalloproteinase (MMP)-1and MMP-9 by the stage of diabetic retinopathy differed significantly among groups. Post hoc analysis showed a significant difference in MMP-1 concentrations between the mild NPDR (non-proliferative diabetic retinopathy) group and the PDR (proliferative diabetic retinopathy) group. MMP-9 concentrations differed significantly between the mild NPDR group and the PDR group; and between the moderate or severe NPDR group and the PDR group.

MMP-9 concentrations differed significantly between the mild NPDR group (27 ± 24 pg/mL) and the PDR group (157 ± 90 pg/mL, p < 0.001); and between the moderate or severe NPDR group (56 ± 32 pg/mL) and the PDR group (p < 0.001). There was no significant difference between the mild NPDR and moderate or severe NPDR group (p = 0.091). The p-values were slightly different in box plots where the median values are compared instead of the mean values (Fig 2).

### DME subgroup analysis by PRP-treatment

**Fig 3:**
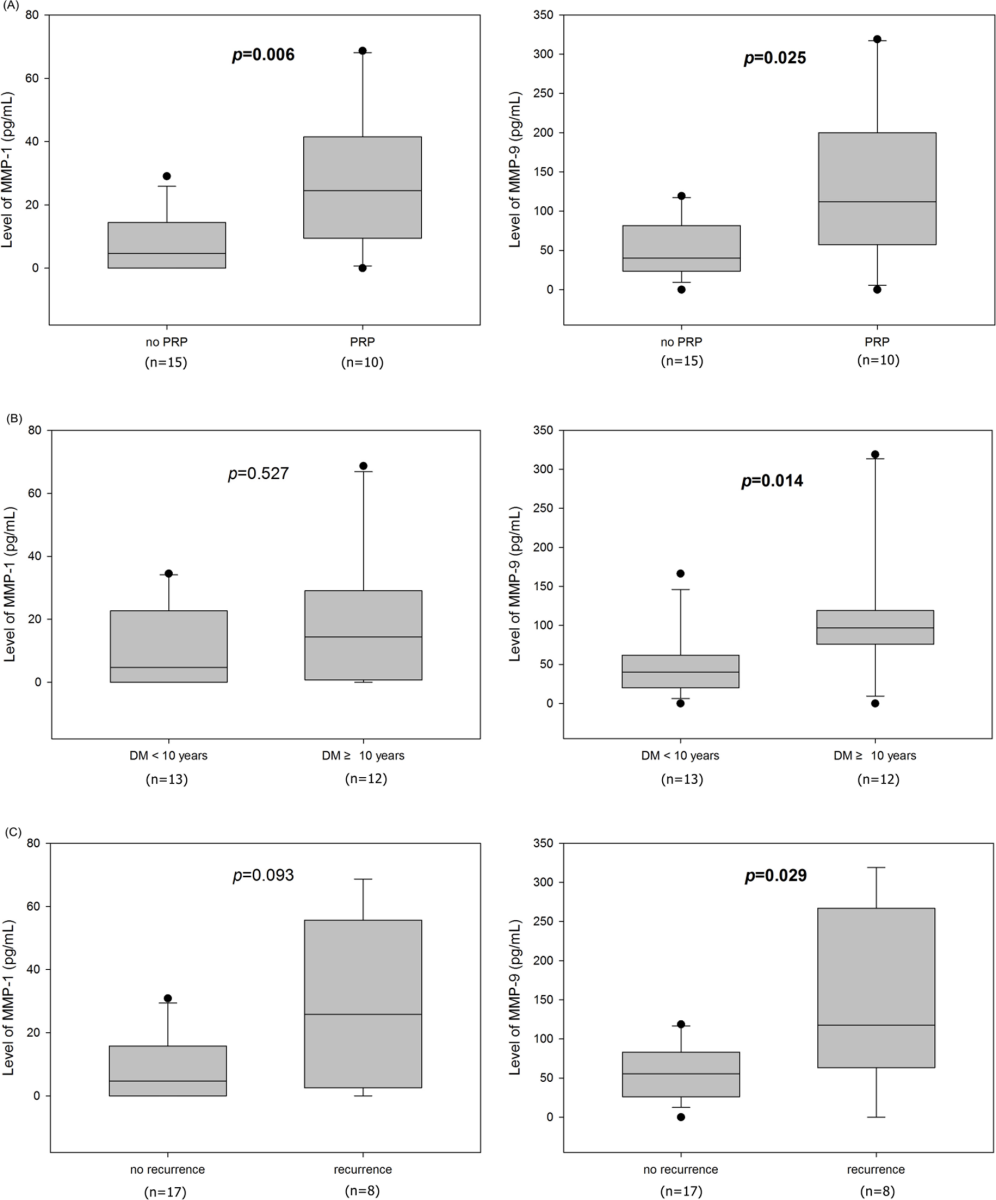
(A) Both metalloproteinase (MMP)-1 and MMP-9 concentrations were significantly higher in pan-retinal photocoagulation (PRP)-treated eyes. (B) MMP-9 concentration was significantly higher in DM eyes with diabetes ≥ 10 years. But there was no significant difference in level of MMP-1 between the two groups. (C) The eyes showed recurrence within 3 months of diabetic macular edema had significantly higher MMP-9 levels than non-recurrent eyes. But there was no statistical difference between the two groups in expression of MMP-1.

Both MMP-1 and MMP-9 concentrations were significantly higher in PRP-treated eyes (n = 10) compared to non- PRP-treated eyes (n = 15), (MMP-1: 8 ± 10 pg/mL versus 28 ± 23 pg/mL; MMP-9: 52 ± 37 pg/mL versus 133 ± 104 pg/mL, p = 0.005, p = 0.023, respectively) (Fig 3(A)).

### DME subgroup analysis by DM duration

The DM eyes were classified as those with diabetes<10 years (n = 13) and those with diabetes≥10 years (n = 12). There was no significant difference in MMP-1 between the two groups (12 ± 13 pg/mL versus 20 ± 23 pg/mL, p = 0.538), whereas MMP-9 differed significantly between the two groups (52 ± 45 pg/mL versus 120± 96, p = 0.011) (Fig 3(B)).

### DME subgroup analysis by recurrence within 3 months

Recurrence within 3 months of DME occurred in eight eyes. These eyes had significantly higher MMP-9 levels than non-recurrent eyes (145 ± 113 versus 56 ± 37 pg/mL, p = 0.027), but no significant difference was evident in MMP-1 levels (10± 10 pg/mL versus 28 ± 26 pg/mL, p = 0.097) (Fig 3(C)).

### The correlation analysis of concentration of MMPs with various factors

Spearman’s correlations were sought among age, axial length, and DM duration; between HbA1C and MMP levels; and between age and MMP levels. The MMP-1 level correlated significantly with age (r = -0.48, p = 0.01). The MMP-9 level correlated significantly with both axial length (r = -0.59, p < 0.01) and DM duration (r = 049, p = 0.01). The HbA1C level did not correlate with MMP levels (Table 2).

**Table 2:** The r-values (the rho values) derived by Spearman’s rank correlation (p-values, two-sided).

	Age	Axial length	DM duration	HbA1C level	MMP-1 level	MMP-9 level
Age		-0.20 (0.35)	0.04 (0.86)	0.15 (0.58)	-0.48 (0.01)	-0.18 (0.38)
Axial length	-0.20 (0.35)		-0.13 (0.56)	0.36 (0.18)	-0.17 (0.44)	-0.59 (<0.01)
DM duration	0.04 (0.86)	-0.13 (0.56)		0.52 (0.05)	0.23 (0.26)	0.49 (0.01)
Hb1AC level	0.15 (0.58)	0.36 (0.18)	0.52 (0.05)		0.20 (0.48)	0.25 (0.38)
MMP-1 level	-0.48 (0.01)	-0.17 (0.44)	0.23 (0.26)	0.20 (0.48)		0.63 (<0.01)
MMP-9 level	-0.18 (0.38)	-0.59 (<0.01)	0.49 (0.01)	0.25 (0.38)	0.63 (<0.01)	

MMP, matrix metalloproteinase; HbA1C, glycated hemoglobin

## Discussion

Most studies have either measured vascular MMP levels in type 1 DM patients or been in vitro experiments[11–15, 32–34]. Although some studies have reported increased ocular MMP levels in diabetic patients, they did not report these levels in different types of patients classified according to DR stage, DM duration, PRP treatment, or degree of myopia [8, 16]. To the best of our knowledge, the present study is the first to characterize factors that may affect MMP-1 and MMP-9 levels in DME patients.

MMP-1 and MMP-9 concentrations in the anterior chamber of DR patients were increased, suggesting that ECM remodeling may be more active in DM patients than in patients without DM. This change was prominently found in patients with PDR and in proportion to the duration of DM.

Flaxel et al. reported that in cultured retinal pigment epithelial cells, MMP-2 and MMP-3 secretion increased to twice that of control values after PRP[35]. However, Sanchez et al. reported that proMMP-9 activity was significantly decreased in vitreous samples from patients with PDR after PRP[36]. In the present study, concentrations of MMPs in the aqueous humor were higher in patients with PRP. Although these results imply that there is increased ECM remodeling in PRP patients, the results may be confounded by other variables such as the stage of DR or the PRP status. We therefore analyzed PRP-treated patients according to the DR stage, to remove possible confounding variables, but the small sample size made it impossible to reach any significant conclusions. Additional studies involving larger cohorts with close follow-ups are necessary to confirm our results.

Axial myopia has a protective association with DR[37, 38]. There are several possible explanations for this relationship. First, decreased vascular diameters in myopic eyes may play a protective role in this association, because DR progression is associated with increased ocular blood flow[39–42]. Second, the dilution effects of cytokines such as transforming growth factor-beta may increase the ocular volume in axial myopia[7]. Third, posterior vitreous detachment (PVD) and vitreous syneresis, frequently present in myopia, may lower the risk of neovascularization progression[43–45]. This protective effect could be associated with the removal of vitreous scaffolds and improved oxygenation as a result of PVD[38, 46, 47] and MMP-9 has been associated with PVD[20]. Fourth, chorioretinal thinning in myopia may reduce oxygen demand and make it easier for oxygen to diffuse into the vitreous[38, 46]. We found that MMP-9 concentrations in the aqueous humor decreased with axial length elongation. In general, our results support these previous theories; however, we did not confirm the effects of PVD on MMP concentration in our study.

We found less significant differences in MMP-1 levels than in MMP-9 levels, although we did find significant differences in MMP-1 concentrations between the DME group and the control group and between the mild NPDR group and the PDR group. In animal model studies, MMP-9 has been shown to have important roles in DR progression to PDR, by facilitating apoptosis of the retinal capillary endothelium and altering tight junctions of the BRB[16, 17, 48]. Studies in humans have also shown significantly increased concentrations of MMP-9 in the aqueous humor[8]. MMP-1 and interstitial collagenase also have roles in extracellular matrix remodeling. They are found mainly in tissues with abundant collagens, such as the macrovascular structure, adipocytes, and the skin of animals and humans[49–52]. The results of the present study suggest that MMP-1 may have a less important role than MMP-9 in inducing DM complications in retinas with a microvascular structure and little connective tissue. A previous study also showed no significant differences in level of MMP-1 between DME and a control group[8].

Our study had several limitations. First, the small number of patients limited our results, and further studies enrolling a larger number of subjects are warranted in the future. Second, the retrospective cross-sectional design of our study made it difficult to identify causal relationships. A longitudinal study such as a randomized controlled trial is needed to confirm the causal relationships of MMP and DR.

In summary, compared to controls, the concentrations of MMP-9 and MMP-1 were increased in the eyes of DR patients, suggesting that MMP-9 and MMP-1 may be involved in the pathogenesis of DME. Furthermore, MMP-9 may have a more important role in the pathogenesis. Additional studies involving larger cohorts are necessary to confirm the results of this study and to show that suppression of MMP levels can reduce the complications of DME. Such studies should increase our understanding of the pathogenesis of MMPs in DR, and may provide a basis for therapeutic approaches to the treatment of DM patients.

## Supporting Information

S1 Text Matrix metalloproteinase-1 and matrix metalloproteinase-9 in the aqueous humor of diabetic macular edema patients. The raw data of this study.
(XLSX)

S1 Fig standard curves. The standard curves of MMPs of this study.
(JPG)
