# Supplementary figures and images for: Matrix Metalloproteinase-1 and Matrix Metalloproteinase-9 in the Aqueous Humor of Diabetic Macular Edema Patients
Source: PLoS One. 2016 Jul 28;11(7):e0159720. doi: 10.1371/journal.pone.0159720 (PMC4965102; doi:10.1371/journal.pone.0159720)

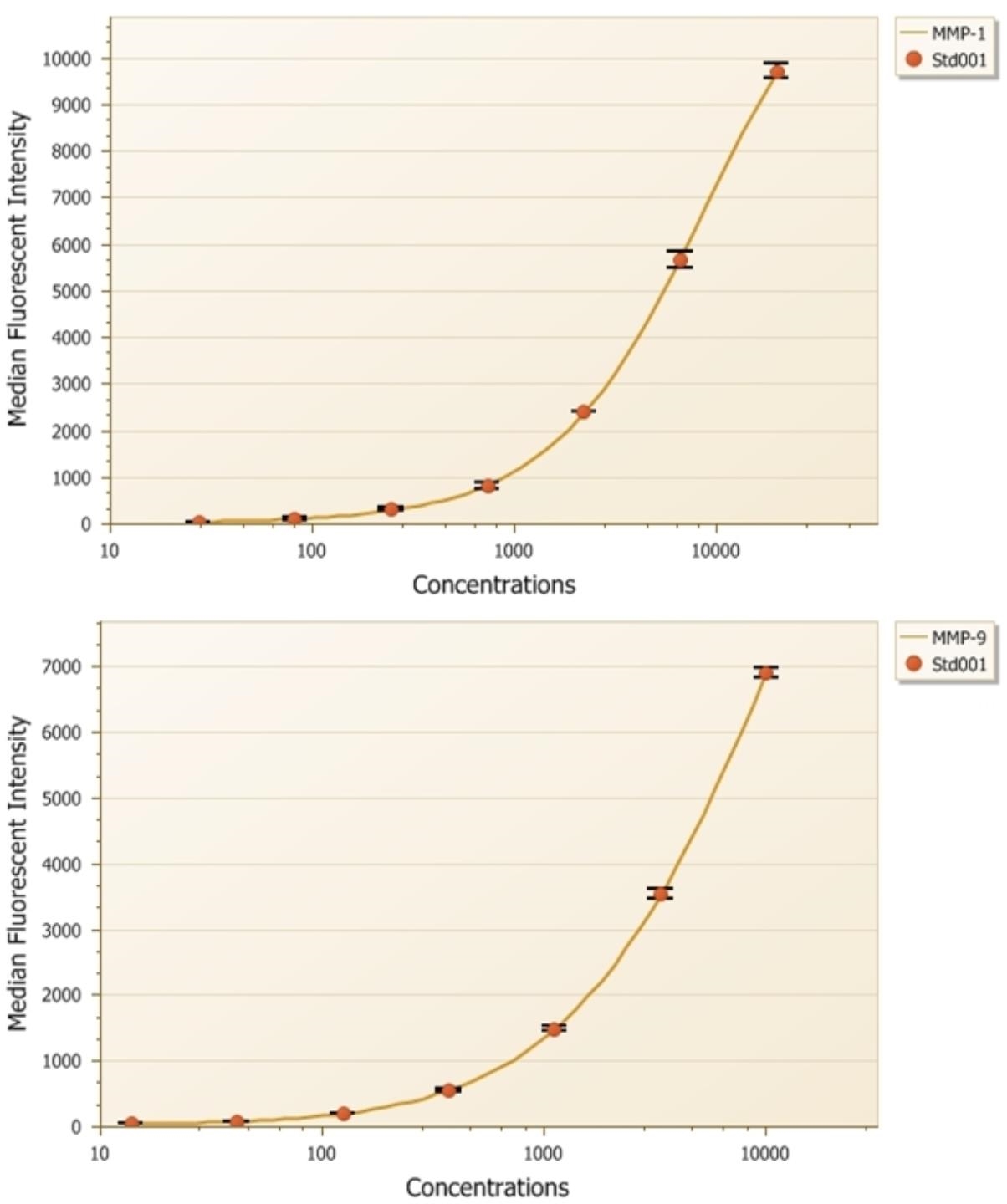

Supplement: S1 Fig — The standard curves of MMPs of this study. (JPG) [file pone.0159720.s002.jpg]
